# Modulation of Microglial Cell Fcγ Receptor Expression Following Viral Brain Infection

**DOI:** 10.1038/srep41889

**Published:** 2017-02-06

**Authors:** Priyanka Chauhan, Shuxian Hu, Wen S. Sheng, Sujata Prasad, James R. Lokensgard

**Affiliations:** 1 Neurovirology Laboratory, Department of Medicine, University of Minnesota Medical School, Minneapolis, Minnesota, USA

## Abstract

Fcγ receptors (FcγRs) for IgG couple innate and adaptive immunity through activation of effector cells by antigen-antibody complexes. We investigated relative levels of activating and inhibitory FcγRs on brain-resident microglia following murine cytomegalovirus (MCMV) infection. Flow cytometric analysis of microglial cells obtained from infected brain tissue demonstrated that activating FcγRs were expressed maximally at 5 d post-infection (dpi), while the inhibitory receptor (FcγRIIB) remained highly elevated during both acute and chronic phases of infection. The highly induced expression of activating FcγRIV during the acute phase of infection was also noteworthy. Furthermore, *in vitro* analysis using cultured primary microglia demonstrated the role of interferon (IFN)γ and interleukin (IL)-4 in polarizing these cells towards a M1 or M2 phenotype, respectively. Microglial cell-polarization correlated with maximal expression of either FcγRIV or FcγRIIB following stimulation with IFNγ or IL-4, respectively. Finally, we observed a significant delay in polarization of microglia towards an M2 phenotype in the absence of FcγRs in MCMV-infected Fcer1g and FcgR2b knockout mice. These studies demonstrate that neuro-inflammation following viral infection increases expression of activating FcγRs on M1-polarized microglia. In contrast, expression of the inhibitory FcγRIIB receptor promotes M2-polarization in order to shut-down deleterious immune responses and limit bystander brain damage.

Chronic neuro-inflammation is a major worldwide health problem. It has been suggested that hyper-immune responses against injury or infectious insult can accelerate the onset and progression of neurodegenerative diseases^1^. To better understand the contribution of inflammation associated with chronic neurodegeneration, we investigated neuroimmune responses during murine cytomegalovirus (MCMV)-induced encephalitis. In humans, cytomegalovirus (CMV) is the leading cause of birth defects due to an infectious agent in the United States^2^. Viral infection of the brain induces a typical innate immune response, driven by microglia^3^,^4^. We have previously established that the primary target cells for MCMV infection within the brain are neural stem cells and infection spreads to astrocytes in highly immunosuppressed hosts^5^,^6^,^7^,^8^. While microglial cells do not themselves support productive viral infection, they do respond to inflammatory mediators produced during viral infection. For example, infected astrocytes generate chemokines such as MCP-1 and IL-8 which recruit antiviral cytokine-producing microglial cells to foci of infection. These activated microglial cells function as sensors for infection and produce cytokines such as, TNF-α and IL-6, as well as additional chemokines to limit viral replication and spread. Hence, MCMV brain infection stimulates microglial cell-driven proinflammatory cytokine and chemokine production which precedes the presence of brain-infiltrating systemic immune cells to control the viral infection. Microglial cells can adopt an activated state with upregulation of FcγRs which clear invading pathogens by triggering antibody dependent cell cytotoxicity (ADCC), phagocytosis, and release of inflammatory mediators; as well as activating other biological sequelae associated with antibody dependent immunity^9^,^10^. To prevent neuronal damage due to exacerbated immune responses, this microglial cell activation needs to be controlled through inhibitory pathways. Hence, it is imperative to maintain the appropriate level of inflammation by striking a balance between activating and inhibitory signals.

FcγRs are found on most cells of the hematopoietic lineage and mediate both high- and low-affinity binding to IgG^11^. FcγRs for IgG couple humoral and cellular immunity by directing the interaction of immune complexes with effector cells^11^. Two broad classes of these receptors have been described: those that activate effector cell responses and those that inhibit^12^,^13^,^14^,^15^. In mice, there are three activating FcγRs (FcγRI, FcγRIII, and FcγRIV) and one inhibitory FcγR (FcγRIIB)^15^. Macrophages and neutrophils express the high-affinity receptor, FcγRI, that cross links to monomeric IgG and mediates ADCC as well as phagocytosis^16^. FcγRllB functions as an inhibitory receptor on B cells while on cells of the myeloid lineage and on platelets, FcyRllB triggers ADCC, phagocytosis, and the release of inflammatory mediators after cross-linking with immune complexes^17^,^18^. FcyRlll is restricted in its expression to natural killer cells, macrophages, neutrophils, and mast cells^19^. It is the only FcγR found on NK cells, mediating all the antibody-dependent responses. FcγRIV expression is restricted to myeloid lineage cells and it binds to IgG2a and IgG2b with intermediate affinity^20^. Hence, different cell types are involved in the regulation of FcγRs.

Activating FcγRs transduce signal activation upon crosslinking by IgG through immunoreceptor tyrosine-based activation motif (ITAM) sequences, usually found on the common γ chain subunit. Activation responses are dependent on the sequential activation of members of the src and syk kinase families, resulting in the recruitment of potent signaling molecules such as PI3 kinase (PI3K) and protein kinase C (PKC)^14^,^20^. On the other hand, inhibitory signals are transduced upon phosphorylation of an immunoreceptor tyrosine-based inhibitory motif (ITIM) sequence found in the cytoplasmic domain of the inhibitory FcγRIIB receptor upon co-crosslinking to an ITAM-containing receptor. This results in the recruitment of the SH2-containing inositol polyphosphate phosphatase (SHIP) and the hydrolysis of PI3K products such as PIP3, leading to the termination of ITAM-initiated activation^21^.

Brain-resident microglial cells, which are pivotal to pathogen detection and initiation of innate neuroimmune responses, co-express activating and inhibitory FcγRs^22^,^23^,^24^. Invading pathogens undergo opsonization with immunoglobulins and microglia recognize these opsonized pathogens through interaction with their cognate FcγRs. Hence, the downstream effector functions are determined by (i) threshold of cellular activation by coupling of immune complexes to the FcγRs and (ii) the relative ratio of these opposing Fcγ receptor molecules. Moreover, in response to insult or injury, microglia mediate multiple facets of neuro-inflammation, including cytotoxic responses, injury resolution, immune regulation, and immunosuppression^25^. Modulation of microglial activation is an appealing strategy employed by the host to promote pathogen clearance, as well as to protect from exacerbated immune responses^26^,^27^. The responding microglia can exist broadly in two different states^28^. The first is a classically activated state (M1), which is typified by the production of pro-inflammatory cytokines and reactive oxygen species; while the second is a state of alternative activation (M2), in which microglia take up an anti-inflammatory phenotype to clear debris and promote repair^29^,^30^,^31^.

We have previously demonstrated that MCMV infection of the central nervous system (CNS) triggers accumulation and persistence of B-lymphocyte lineage cells within the brain. We also showed the presence of MCMV-specific antibody secreting cells within the infiltrating leukocytes that co-localize with IgG or IgM^32^. In this study, we first determined the relative ratios of both activating as well as inhibitory FcγRs on microglial cells following MCMV brain infection. Further, we demonstrated the effect of IFNγ and IL-4 in polarizing microglia to M1 and M2 phenotype, respectively; and analyzed expression of activating as well as inhibitory FcγRs on the polarized microglia. Lastly, we demonstrated the role of FcγRs in microglial switching to M2 phenotype by employing mice deficient in either activating or inhibitory FcγRs.

## Results

### *In vivo* model of chronic neuro-inflammation following MCMV-induced encephalitis

**Figure 1:**
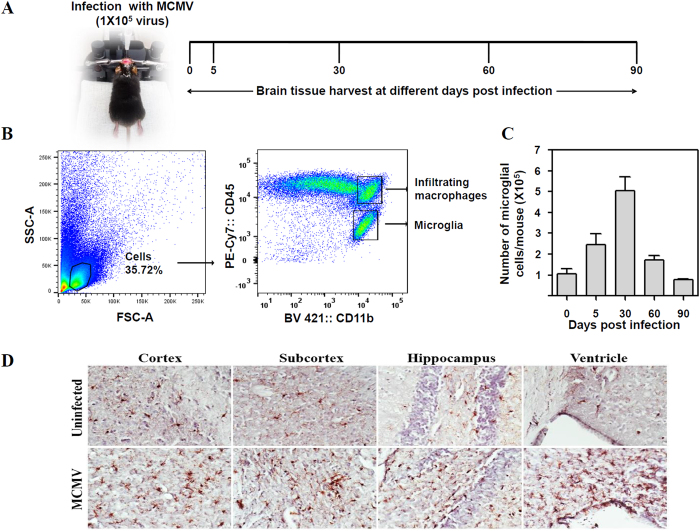
Chronic neuro-inflammation following MCMV-induced encephalitis. Mice were infected with 1 × 10^5^ TCID_50_ units (in 10 μl) of MCMV. One group of mice was not infected with MCMV. At 0, 5, 30, 60 and 90 dpi, mice were euthanized and brain tissues were harvested to isolate brain mononuclear cells (BMNCs). (**A**) Treatment and sampling schedule. (**B**) The representative flow cytometric dot plots to identify microglial cells. BMNCs were first gated on their forward and side scatter properties followed by gating on CD45 and CD11b. Gating on the CD45^int^CD11b^hi^ population identified microglial cells. (**C**) The number of brain resident microglial cells post infection. Counts were acquired on a FACS LSR II H4760 flow-cytometer (using FACS Diva software) and analyzed using FlowJo software (Tree Star, USA). Data presented are mean ± SE of two experiments with 4–6 mice per time point. (**D**) Immunohistochemical staining of brain sections demonstrating persistence of microglial cells (brown) in the cortex, subcortex, hippocampus and ventricle regions of uninfected and MCMV-infected mice at 30 dpi.

To establish viral brain infection, we performed intracerebroventricular inoculation of mice with MCMV as described in the Methods. Mice were infected with 1 × 10^5^ TCID_50_ units in 10 μl; and tissues were harvested at 5, 30, 60, and 90 dpi (Fig. 1A). One group of mice remained uninfected. At each time point, mice were euthanized and brains were harvested to isolate mononuclear cells for flow cytometric analysis. Cells were first gated on their forward and side scatter characteristics followed by gating on CD45 and CD11b. Gating on the CD45^int^CD11b^hi^ population identified the microglial cell population (Fig. 1B). This technique allows for differentiation between brain-resident microglia and brain-infiltrating macrophages which are identified as CD45^hi^CD11b^hi^, as shown in Fig. 1B^33^. A previous study from our laboratory has demonstrated that microglia undergo active proliferation (as Ki67 positive) in response to MCMV brain infection^34^. Therefore, the total number of microglial cells was enumerated and it was established that their number increased until 30 dpi, after which there was a decline (Fig. 1C). Moreover, immunohistochemical staining for Iba-1 (a microglial cell marker) in brain sections from MCMV-infected animals displayed microglial nodules with reactive morphology in the cortex, subcortex, hippocampus, and ventricle regions of the brain at 30 dpi (Fig. 1D).

### Impact of viral infection on the cytokine milieu and microglia FcγR expression

**Figure 2:**
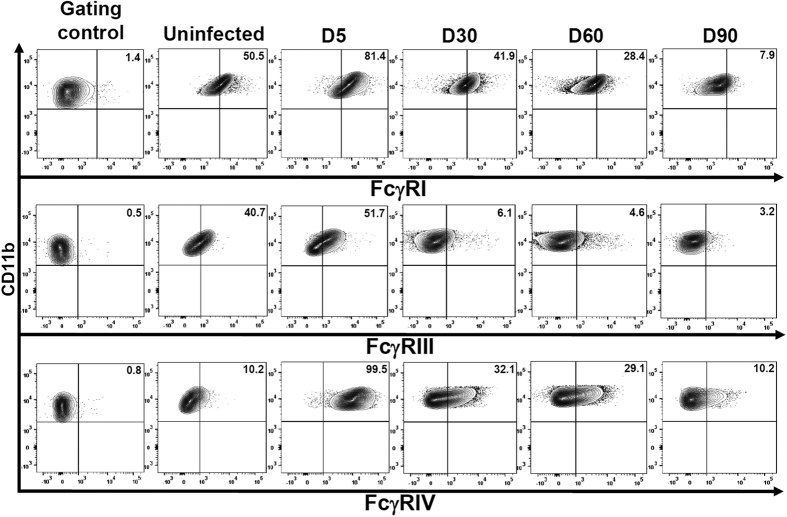
Impact of viral infection on microglial cell activating FcγR expression. BMNCs were extracted from brains of uninfected and MCMV-infected mice at 0, 5, 30, 60 and 90 dpi. Microglia were first identified as CD45^int^CD11b^hi^ cells and subsequently stained for activating FcγRs (FcγRI, FcγRIII and FcγRIV). Flow cytometric contour plots are representative of two separate experiments using 4–6 mice per time point. Gating control is the Fcer1g strain of mice that lacks activating FcγRs. Data presented show mean frequency of microglial cells expressing each FcγR at the corresponding time point.

**Figure 3:**
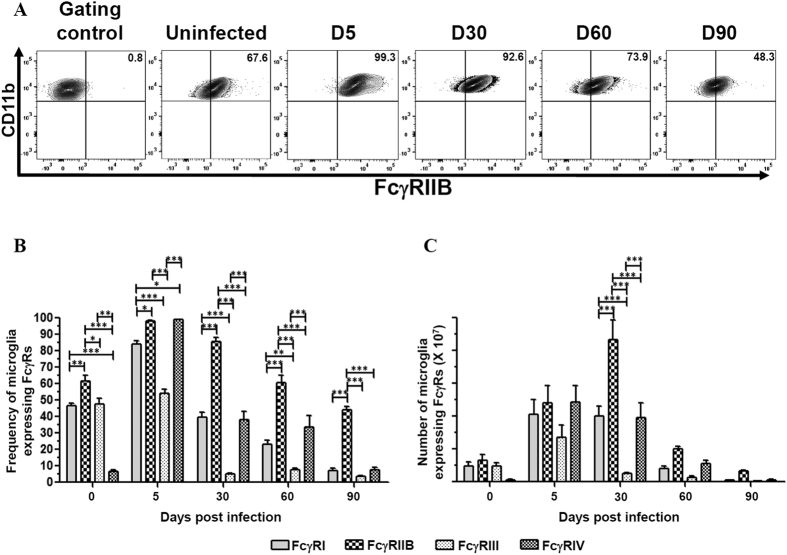
Impact of viral infection on microglial cell inhibitory FcγR expression. BMNCs were extracted from brains of uninfected and MCMV-infected mice at 0, 5, 30, 60 and 90 dpi. Microglia were first identified as CD45^int^CD11b^hi^ cells and subsequently stained for inhibitory FcγR (FcγRIIB). (**A**) Flow cytometric contour plots are representative of two separate experiments using 4–6 mice per time point. Gating control is the FcgR2b strain of mice that lacks inhibitory FcγR. Data presented show mean frequency of microglial cells expressing inhibitory FcγR at the corresponding time point. (**B**) Frequency of microglial cells expressing FcγRs were calculated based on flow cytometric analysis from MCMV infected brain at 0, 5, 30, 60 and 90 dpi. (**C**) Absolute numbers of microglial cells expressing FcγRs observed at the indicated time points. Pooled data are presented as mean ± SE of two experiments using 4–6 mice per time point. The data was analyzed using two-way analysis of variance (ANOVA) followed by Bonferroni post-tests (*p < 0.05, **p < 0.01, and ***p < 0.001).

MCMV infection-induced neuroinflammation results in the production of various chemokines and cytokines by astrocytes and microglial cells. The outcome of brain infection as well as microglial cell polarization is largely dependent on the type of cytokines present within the brain microenvironment. Hence, in this study we investigated the presence of both pro- and anti-inflammatory molecules generated during the course of infection. We observed that there was a significant increase in production of the pro-inflammatory molecules IFNγ and MHC-II during the acute phase of infection at 5 dpi (***p < 0.001). In contrast, there was an overall increase in expression of the anti-inflammatory molecules IL-4 and TGFβ during both acute and chronic phases of infection (Supplementary Figure 1). Further, we investigated the relative expression of both activating (FcγRI, FcγRIII, and FcγRIV) and inhibitory (FcγRIIB) FcγRs on microglial cells in the inflammatory milieu of infected brains. We infected mice with MCMV intracerebroventricularly and evaluated their relative expression during both the acute (5 dpi) and chronic phases of infection (30, 60, and 90 dpi). One group of animals was treated as mock (uninfected naïve mice at d 0). Flow cytometric analysis of microglial cells obtained from infected brain tissue demonstrated that the activating FcγRs were expressed maximally at 5 dpi. FcγRI was found to be expressed on 81.4% of the cells at 5 dpi, declined by 30 dpi (41.9%), and was expressed on 7.9% of the microglia by 90 dpi (Fig. 2). Similarly, expression of FcγRIII was maximum at 5 dpi (51.7%) following which there was a decline, which varied between 6.1% to 3.2% of the cells. Interestingly, highly inducible expression of the activating FcγRIV (99.5%) during the acute phase of infection (5 dpi) was observed, followed by a substantial decline by 90 dpi (10.2%) (Fig. 2). In contrast to the activating FcγRs, the inhibitory receptor (FcγRIIB) remained highly elevated during both the acute (i.e at 5 dpi, 99.3%) and chronic phase of infection [i.e., 30 (92.6%), 60 (73.9%), and 90 (48.3%) dpi], (Fig. 3A). When the percentage and the number of microglial cells expressing FcγRIIB was compared with the activating FcγRs, a significantly higher expression of the inhibitory FcγR was observed during chronic phase of infection (***p < 0.001), (Fig. 3B and C).

### Microglial cell polarization following IFNγ and IL-4 treatment

Several studies have identified the role of pro- and anti-inflammatory cytokines in polarizing macrophages and microglial cells into distinct activation states^25^,^31^,^35^. In this study, we employed IFNγ and IL-4 as potent M1/M2 polarizing stimuli. Phenotypic markers useful to identify microglial cells which were M1-polarized included iNOS, tumor necrosis factor (TNF)-α, and CD86. Likewise, markers useful for quantifying M2-polarized microglia included Arginase-1, E-cadherin, and CD206. So, we exposed primary murine microglial cells to either IFNγ or IL-4 for either 6 h or 24 h, and assessed mRNA expression indicative of M1/M2 markers. Prototypical pro-inflammatory stimulation with IFNγ increased mRNA expression of all the studied cytotoxic M1 markers (Supplementary Figure 2A–C). Likewise, treatment with the prototypical anti-inflammatory cytokine IL-4 increased mRNA expression of M2 phenotype markers (Supplementary Figure 2D–F). Thus, IFNγ treatment was demonstrated to polarize the microglial cells to an M1 phenotype, while IL-4 stimulation switched the cells to an M2 phenotype.

### Expression of FcγRs on polarized microglial cells *in vitro*

**Figure 4:**
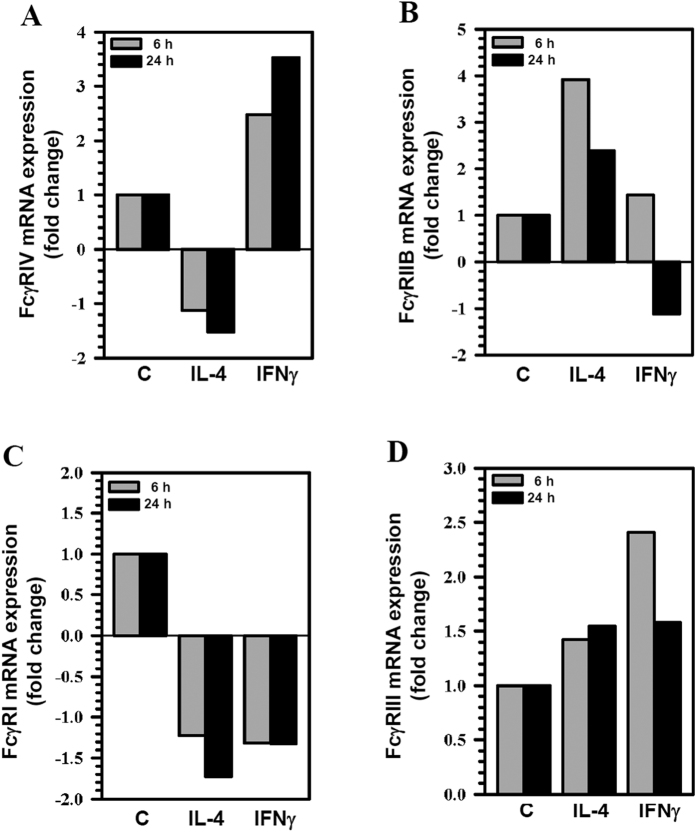
Differential mRNA expression of FcγRs on microglial cells polarized *in vitro.* Primary murine microglial cells were either unstimulated (**C**) or stimulated with IL-4 or IFNγ for 6 h and 24 h. The cDNA synthesized from 1 μg RNA was amplified and fold change of mRNA expression of FcγRs (**A**) FcγRIV; (**B**) FcγRIIB; (**C**) FcγRI and (**D**), FcγRIII relative to unstimulated control was quantified using the 2^−∆∆Ct^ method and normalized to the housekeeping gene HPRT. Data shown are representative of two experiments.

To characterize how IFNγ- or IL-4-polarization altered expression of activating as well as inhibitory FcγRs on microglia, we assessed FcγR expression levels of primary microglial cells stimulated with either IFNγ or IL-4. *A*nalysis using qRT-PCR demonstrated enhanced mRNA expression of the activating receptor, FcγRIV (2.5 fold at 6 h and 3.5 fold at 24 h) following stimulation with IFNγ (Fig. 4A). In contrast, we observed a corresponding increased mRNA expression of the inhibitory receptor, FcγRIIB (3.9 fold at 6 h and 2.4 fold at 24 h), following stimulation with IL-4 (Fig. 4B). We did not observe any significant differences in the mRNA expression of FcγRI and FcγRIII (Fig. 4C and D).

### FcγRs and microglial cell polarization following viral infection

**Figure 5:**
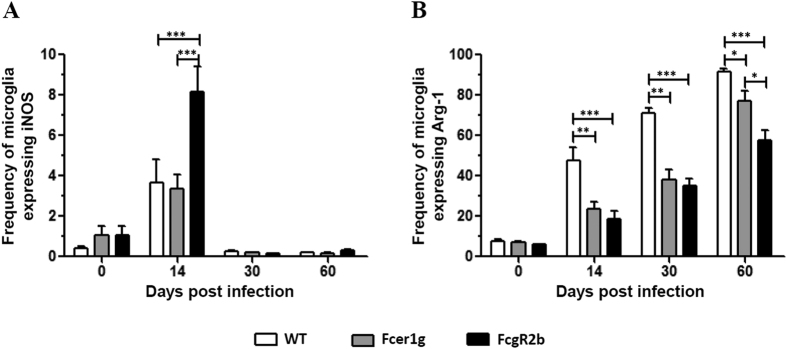
Effect of viral infection on the frequency of microglia expressing iNOS and Arg-1 in C57BL/6, Fcer1g and FCgR2b. (**A**) Frequency of microglial cells expressing iNOS were calculated based on flow cytometric analysis from MCMV infected brain at 0, 14, 30 and 60 dpi. (**B**). Frequency of microglial cells expressing Arg-1 observed at the indicated time points. Pooled data presented are mean ± SE of two experiments using 4–6 mice per time point. The data was analyzed using regular two-way analysis of variance (ANOVA) followed by Bonferroni post-tests (*p < 0.05, **p < 0.01, and ***p < 0.001.

We next investigated if FcγRs play a role in switching microglial cells from an M1- to M2-polarized state. In these studies, C57BL/6 (WT), Fcer1g KO (mice deficient in the γ chain subunit of activating FcγRs), and Fcgr2b KO (mice deficient in FcγRIIB) mice were infected with MCMV and the expression of iNOS and Arg-1, the two most prominent M1/M2 differentiating markers, was analyzed on microglial cells at various dpi. Following viral infection, a significant increase in the frequency of microglia expressing iNOS was found in FcgR2b KO mice (8.07%) when compared with either WT (3.68%) (***p < 0.001) or Fcer1g KO mice (3.08%) (***p < 0.001) at 14 dpi (Fig. 5A). This finding demonstrates that microglia remained in a prolonged, activated pro-inflammatory M1 state in the absence of the inhibitory FcγR. Likewise, when Arg-1 expression was monitored, we observed an increase in the frequency of microglia expressing this M2 marker (7.43% at 0 dpi, 47.7% at 14 dpi, 71.0% at 30 dpi, and 91.5% at 60 dpi) in the WT mice (Fig. 5B). We also observed substantial increase in the frequency of microglia expressing Arg-1 in both Fcer1g KO (6.85% at 0 dpi, 20.1% at 14 dpi, 38.3% at 30 dpi, and 77.0% at 60 dpi) and FcgR2b KO (6.0% at 0 dpi, 18.5% at 14 dpi, 35.2% at 30 dpi, and 57.5% at 60 dpi) animals with increasing dpi. However, expression of this M2 marker on microglial cells in both Fcer1g KO and FcgR2b KO animals was significantly lower when compared to the WT mice (Fcer1g vs WT; **p < 0.01 at 14 and 30 dpi, *p < 0.05 at 60 dpi), (FcgR2b vs WT; ***p < 0.001 at 14, 30 and 60 dpi), (Fig. 5B). At 60 dpi, we observed a significantly lower frequency of microglial cells expressing Arg-1 in FcgR2b KO mice (57.5%) when compared with WT (91.51%) (**p < 0.001) and Fcer1g KO mice (77.04%) (*p < 0.05), (Fig. 5B). Thus, in the absence of the inhibitory receptor FcγRIIB, there was reduced polarization of microglia into an M2 phenotype.

## Discussion

HCMV is generally acquired as an asymptomatic, subclinical infection in immune competent persons^36^. However, it is also the most common infectious cause of congenital birth defects. HCMV can establish latency and persistence in monocyte precursors and diverse populations of tissue stromal cells^37^. It is clear that the virus can rapidly reactivate from this systemic latency upon immunosuppression. Hence, constant immune surveillance is required to keep persistent infection in check. Replication of cytomegaloviruses is highly species-restricted and, therefore, no natural animal model exists for examining HCMV pathogenesis. Consequently, CMV infection has been studied extensively in the mouse model, a model which not only provides several advantages due to the availability of genetically characterized inbred strains, but also exhibits conserved viral tissue tropism and temporal regulation of gene expression. Therefore, HCMV and MCMV display similar pathogenesis^38^. During both infections, the immune system plays a crucial role not only in controlling the spread of viral infection but also in stimulating the shift from productive viral infection to a state of viral persistence^39^. It has been demonstrated that soluble mediators such as, cytokines and chemokines produced by various immune cells inhibit viral replication in various cell types. In addition, CMV-specific T lymphocytes protects against the lethal effect of viral infection. However, there is also evidence for a dual role of immune responses in shifting the state of viral persistence to productive infection (i.e. reactivation of viral infection). Hence, during CNS viral infections, a complex multi-directional interaction between cytokines, chemokines, and cellular machinery of the immune system determine the outcome of infection, resulting in either resolution or disease.

Innate and adaptive immune responses have evolved selective pathways to resolve microbial infections while simultaneously preventing these same pathways from triggering unnecessary collateral tissue damage. This use of selective immune pathways is seen at many levels, from the mechanisms by which dendritic cells induce both tolerogenic and immunogenic responses, to the pathways that give rise to selective expression of activating or inhibitory signals in response to specific pathogens^40^,^41^. Disturbances in this system, either due to enhanced activating or decreased inhibitory signals, may lead to excessive immune activation resulting in tissue damage, induction of autoimmune disease, and chronic inflammation^42^. This balance is achieved by the integration of inhibitory and activating signals, which are delivered by pairs of cell surface receptors.

The regulation of IgG activity through cellular FcγRs on various immune cells represents another example of polarization of immune function in response to specific challenges^15^,^43^. This is not only relevant for the regulation of antibody-mediated effector functions through innate immune effector cells, but also for the regulation of B-cell activation and antibody production^13^. Immunoglobulin FcγRs constitute a family of hematopoietic cell-surface molecules that include receptors which mediate both high- and low-affinity binding to IgG thereby, either stimulating or inhibiting cellular responses upon crosslinking to antibody-antigen complexes^11^,^16^,^44^. Therefore, we investigated the *in vivo* expression of these activating, as well as inhibitory, FcγRs on microglial cells at various times post MCMV-induced encephalitis. The results obtained in this study, clearly demonstrate the increased expression of activating FcγRs to promote pathogen clearance during acute phase of infection.

The FcγR system has evolved distinct receptors displaying selectivity for IgG subclasses^13^,^20^. IgG1 binds exclusively with low affinity (0.3 × 10^6^ M^−1^) to the activation receptor FcγRIII, whereas IgG2a binds with low affinity (0.7 × 10^6^ M^−1^) to FcγRIII and with 40-fold higher affinity to FcγRIV^20^. These distinct binding affinities for the IgG subclasses to FcγRs account for their differential protective and pathogenic activities *in vivo*. Several studies have suggested that IgG2a is the most potent subclass in mediating protection and has a preferential dependence on FcγRIV activation^20^,^45^,^46^. This may explain the increased expression of FcγRIV on microglia during the acute phase of infection. It has been previously shown that IFNγ is a strong stimulus to induce Th1 activation^47^. In our study, we observed significant enhancement in the expression of FcγRIV on microglial cells following stimulation with IFNγ. This suggests that Th1 activation induces both IgG2a expression and its activation receptors, FcγRIV, thereby amplifying the role of this subclass in mediating effector responses *in vivo*.

We also observed preferential expression of the inhibitory receptor FcγRIIB on microglial cells during chronic infection, possibly to prevent hyper-immune responses and subsequent bystander brain damage. Several studies have demonstrated that FcγRIIB acts as a general negative regulator of immune complex triggered activation *in vivo*^42^,^48^,^49^. Mast cells from FcγRII^−/−^ mice are highly sensitive to IgG-triggered degranulation, in contrast to their wild type counterparts^50^. FcγRIIB-deficient mice exhibited an enhanced passive cutaneous anaphylaxis reaction. Disruption of FcγRIIB by gene targeting resulted in mice with elevated Ig levels in response to both thymus-dependent and thymus-independent antigens, enhanced passive cutaneous anaphylaxis reaction, and enhanced immune complex (IC)-mediated alveolitis^50^. These studies indicate that FcγRIIB physiologically acts as a negative regulator of IC-triggered activation and may function *in vivo* to suppress autoimmunity by regulating both B cell responses and effector cell activation^17^.

At sites of brain inflammation, microglial cell activity is regulated by T-cell derived cytokines and is linked to their polarization into M1 and M2 phenotypes^25^. The M1 phenotype, as marked by the production of iNOS, TNF-α, and CD86 is optimized to facilitate the elimination of intracellular pathogens through the release of Th1 cytokines such as IFNγ^51^. Th2 cytokines such as IL-4, on the other hand, are generally produced in response to chronic infection and may provide a protective mechanism to prevent hyper immune responses and bystander brain damage^31^. We found that microglial cell switching to an M2 phenotype is characterized by increased expression of Arg-1, E-Cadherin, and CD206. Our data was consistent with the observation that IFNγ stimulation drives the microglia towards M1 phenotype and IL-4 stimulation polarizes these cells towards an M2 phenotype. In this study, we investigated the expression of FcγRs on these cytokine-polarized microglia. Using semi-quantitative real-time PCR, we show that IFNγ induced the expression of FcγRIV but did not induce changes in other activating receptors. Likewise, IL-4 also induced a substantial increase in the expression of FcγRIIB, but had no effect on other FcγRs expression.

We next employed knockout mice deficient in either the activating receptors (Fcer1g) or the inhibitory receptor (FcgR2b) and analyzed expression of iNOS and Arg-1, prototypic markers for M1 and M2, respectively. In these experiments, we observed a significant increase in the expression of iNOS in FcgR2b KO mice when compared with WT and Fcer1g KOs demonstrating that microglia remained in an activated pro-inflammatory M1 state in the absence of this inhibitory FcγR. Likewise, when Arg-1 expression was assessed, we observed a significantly lower frequency of microglial cells expressing Arg-1 in FcgR2b KO mice when compared with WT at all the time points of the study. Moreover, at a later time point (60 dpi), we observed a significant decrease in Arg-1 expression in FcgR2b KO when compared with Fcer1g KO animals. Loss of the M2 phenotype in the absence of FcγRIIB suggests a role for this receptor in driving the polarization of microglia towards this phenotype. However, the role of activating Fcγ receptors in driving the microglia towards M2 phenotype can also not be negated. We observed a significant decrease in the frequency of microglia expressing Arg-1 in the Fcer1g KO strain as well, when compared with WT. Recent studies report an unexpected role for FcγRI and FcγRIII in mediating suppressive effects, thereby linking the loss of these suppressive effects with loss of the M2 phenotype in Fcer1g KO mice^52^,^53^.

To conclude, our study demonstrated for the first time the relative expression of activating as well as inhibitory Fcγ receptors specifically on microglial cells post-MCMV brain infection. We also show a role of FcγRs in microglial phenotype switching. The data presented in this study clearly reveal three major findings. First, acute neuroinflammation following MCMV infection increases expression of activating FcγRs, likely to promote pathogen clearance through increased effector cell activation. Secondly, preferential expression of the inhibitory receptor during both acute and chronic infection phases may provide a protective mechanism to prevent hyper-immune responses and subsequent bystander brain damage. Thirdly, we observed a significant delay in the polarization of microglia towards an M2 phenotype in the absence of FcγRs in MCMV-infected mice. Hence, it is evident that the modulation of Fcγ receptors on microglia play a vital role in disease pathogenesis and microglial switching. The results obtained in this study will be useful for further investigations of the role of FcγRs in mediating effector functions by using Fcer1g and FcgR2b strains of mice that lack activating and inhibitory receptors, respectively.

## Methods

### Ethical statement

This study was carried out in strict accordance with recommendations in the Guide for the Care and Use of Laboratory Animals of the National Institutes of Health. The protocol was approved by the Institutional Animal Care and Use Committee (Protocol Number: 1402–31307 A and breeding Protocol Number: 1403-31431 A) of the University of Minnesota. All animals were routinely cared for according to the guidelines of Research Animal Resources (RAR), University of Minnesota. All surgery was performed under Ketamine/Xylazine anesthesia and all efforts were made to ameliorate animal suffering. Animals were sacrificed after isoflurane inhalation, whenever required.

### Virus and growth conditions

RM461, a recombinant MCMV expressing *E. coli* β-galactosidase under the control of the human ie1/ie2 promoter/enhancer^54^ was kindly provided by Edward S. Mocarski (Supplementary Table 1). Viral stocks were passaged in salivary glands of weanling female Balb/c mice to retain their virulence. Virus isolated from the salivary glands was then passaged twice on NIH 3T3 fibroblasts to minimize any carry-over of salivary gland tissue. Infected 3T3 cultures were harvested at 80% to 100% cytopathic effect and subjected to three freeze–thaw cycles. Cellular debris was removed by centrifugation (1000 × g) at 4 °C, and the virus was pelleted through a 35% sucrose cushion (in Tris-buffered saline [50 mM Tris–HCl, 150 mM NaCl, pH 7.4]) at 23,000 × g for 2 h at 4 °C. The pellet was suspended in Tris buffered saline containing 10% heat-inactivated fetal bovine serum (FBS). Viral stock titers were determined on 3T3 cells as 50% tissue culture infective doses (TCID_50_) per milliliter. This sucrose gradient-purified RM461 was used for intracerebroventricular infections of mice.

### Experimental animals

Pathogen free C57BL/6 mice (as wild type (WT) control), Fcer1g mice (Model 583; mice deficient in the γ chain subunit of activating FcγRs) and Fcgr2b mice (Model 580; mice deficient in FcγRIIB) were purchased from Taconic Biosciences, Inc. (Hudson, NY), (Supplementary Table 1). The animals were housed in individually ventilated cages and were provided with food and water *ad libitum* at the RAR facility, University of Minnesota. The knockout strains were equally susceptible as the parental strain to MCMV infection as assessed by viral expression levels of immediate early (IE-1) and early (E-1) mRNAs, using by semi quantitative RT-PCR (Supplementary Figure 3).

### Intracerebroventricular infection of mice

Infection of mice with MCMV was performed as previously described^55^,^56^. Briefly, female mice (8 weeks old) were anesthetized using a combination of Ketamine and Xylazine (100 mg/kg and 10 mg/kg body weight, respectively) and immobilized on a small animal stereotactic instrument equipped with a Cunningham mouse adapter (Stoelting Co., Wood Dale, IL). The skin and underlying connective tissue were reflected to expose reference sutures (sagittal and coronal) on the skull. The sagittal plane was adjusted such that bregma and lambda were positioned at the same coordinates on the vertical plane. Virulent, salivary gland-passaged MCMV RM461 (1 × 10^5^ TCID_50_ units in 10 μl), was injected into the right lateral ventricle at 0.9 mm lateral, 0.5 mm caudal to the bregma and 3.0 mm ventral to the skull surface using a Hamilton syringe (10 μl) fitted to a 27 G needle. The injection was delivered over a period of 3–5 min. The opening in the skull was sealed with bone wax and the skin was closed using 4–0 silk sutures with a FS-2 needle (Ethicon, Somerville NJ).

### Isolation of brain leukocytes and flow cytometric analysis

Mononuclear cells were isolated from the brains of MCMV-infected C57BL/6, Fcer1g and FcgR2b mice using a previously described procedure with minor modifications^57^,^58^,^59^. In brief, whole brain tissues were harvested, (n = 4–6 animals/group/experiment), and minced finely using a scalpel in RPMI 1640 (2 g/L D-glucose and 10 mM HEPES) and digested in 0.0625% trypsin (in Ca/Mg-free HBSS) at room temperature for 20 min. Single cell preparations of infected brains were suspended in 30% Percoll and banded on a 70% Percoll cushion at 900× g for 10 min at 15 °C. Brain leukocytes obtained from the 30–70% Percoll interface were collected.

Following preparation of single cell suspensions, cells were treated with Fc block (anti-CD32/CD16 in the form of 2.4G2 hybridoma culture supernatant with 2% normal rat and 2% normal mouse serum) to inhibit nonspecific Ab binding. In case, when the expression of Fcγ receptors was analyzed, the addition of Fc block was avoided. Cells were then counted using the trypan blue dye exclusion method, and 1 × 10^6^ cells were subsequently stained with anti-mouse immune cell surface markers for 15 min at 4 °C (anti-CD45-PE-Cy7 (eBioscience, San Diego CA), anti-CD11b-BV421 (BioLegend, San Diego CA), anti-FcγRI-BV711 (BioLegend), anti-FcγRIIB-APC (eBioscience), anti-FcγRIII-FITC (R&D Systems Inc., Minneapolis MN) and anti-FcγRIV-PE (BioLegend). Control isotype Abs were used for all fluorochrome combinations to assess nonspecific Ab binding. 10^5^ cells were acquired per sample by using a FACS LSR flow-cytometer (by employing FACS DIVA software). Firstly, viable leukocytes were gated based upon their forward scatter and side scatter characteristics on a BD FACS LSR flow cytometer (BD Biosciences, San Jose CA). The leukocytes were then gated by using CD45-PE-Cy7 and CD11b-BV421 for the selection of microglial population (CD45^int^CD11b^hi^). The gated microglial population was then analyzed for the expression of FcγRs. Data were analyzed using FlowJo software (FlowJo, Ashland, OR).

### Intracellular cytokine staining

To determine the expression of inducible nitric oxide synthase (iNOS) and arginase-1 (Arg-1) by microglia, brain mononuclear cells were harvested as described in previous section. Cells were surface stained using anti-CD45-PE-Cy7 and anti-CD11b-BV421 prior to fixation/permeabilization using cytofix/cytoperm kit (eBioscience). Cells were then stained with anti-iNOS-PE (eBioscience) and anti-Arg-1-FITC (R&D Systems), as recommended by manufacturer’s protocol.

### Primary murine microglial cell cultures

Murine cerebral cortical cells from 1-day-old mice were dissociated after a 30 min trypsinization (0.25%) and plated in 75-cm^2^ Falcon culture flasks in DMEM containing 10% FBS, penicillin (100 U/ml), streptomycin (100 μg/ml), gentamicin (50 μg/ml) and Fungizone^®^ (250 pg/ml). The medium was replenished 1 and 4 d after plating. On d 12 of culture, floating microglial cells were harvested and plated onto 6-well tissue culture plates and incubated at 37 °C. Purified microglial cells were >95% stained positively with Iba-1 antibodies (phenotypic marker of microglia) and <2% stained positively with antibodies specific to glial fibrillary acidic protein (GFAP) (phenotypic marker of astrocytes). Microglial cells were then stimulated with IFNγ (10 ng/ml) or IL-4 (30 ng/ml) and analyzed for the expression of FcγRs and their M1/M2 phenotype.

### Semi-quantitative RT-PCR

Total RNA from primary glial cell cultures, or from brain tissue was extracted using an RNeasy Mini Kit (Qiagen, Valencia, CA) or TRIzol reagent (Invitrogen, Carlsbad, CA), respectively. The cDNA was synthesized from total RNA (1 μg) using Superscript III reverse transcriptase (Invitrogen) and oligo d(T)_12–18_ primers (Sigma-Aldrich, St. Louis, MO). The list of primers employed in the study is tabulated in Supplementary Table 1. PCR was performed with the SYBR Advantage qPCR master mix (ClonTech, Mountain View, CA). The qPCR conditions were: 1 denaturation cycle at 95 °C for 10 s; 40 amplification cycles of 95 °C for 10 s, 60 °C annealing for 10 s, and elongation at 72 °C for 10 s; followed by 1 dissociation cycle (Mx3000 P QPCR System, Stratagene, now Agilent Technologies, La Jolla, CA). The relative expression levels were quantified using the 2^−∆∆Ct^ method^60^ and were normalized to the housekeeping gene hypoxanthine phosphoribosyl transferase (HPRT).

### Immunohistochemistry

Brains were harvested from both uninfected and MCMV-infected animals that were perfused with serial washes of phosphate-buffered saline (PBS), 2% sodium nitrate to remove contaminating blood cells, and 4% paraformaldehyde. Murine brains were subsequently submerged in 4% paraformaldehyde for 24 h and transferred to 25% sucrose solution for 2 d prior to sectioning. After blocking (10% normal goat serum and 0.3% Triton X-100 in PBS) for 1 h at room temperature (RT), brain sections (30 μm) were incubated overnight at 4 °C with rabbit anti-ionized calcium binding adaptor molecule (Iba)1 (2 μg/mL; Wako Chemicals, Richmond, VA). After washing three times with TBS, secondary Ab (goat anti-rabbit IgG biotinylated; Vector Labs, Burlingame, CA) was added for 1 h at RT followed by incubation with ABC (avidin-biotinylated enzyme complex, Vector Labs) solution. The peroxidase detection reaction was carried out using 3,3′-diaminobenziding tetrahydrochloride (DAB; Vector Labs) for several minutes at RT.

### Statistical analysis

One-way analysis of variance (ANOVA) with Tukey’s multiple comparison Test or Two-way ANOVA followed by Bonferroni posttests were employed, as appropriate. Differences were considered significant, when p < 0.05. For statistical analysis and generation of graphs, Prism 5 software (Version 5.01; GraphPad Software Inc., USA) was used.

## Additional Information

**How to cite this article:** Chauhan, P. *et al*. Modulation of Microglial Cell Fcγ Receptor Expression Following Viral Brain Infection. *Sci. Rep.* **7**, 41889; doi: 10.1038/srep41889 (2017).

**Publisher's note:** Springer Nature remains neutral with regard to jurisdictional claims in published maps and institutional affiliations.

## Supplementary Material

Supplementary Figures and Table
