# Supplementary material for: Modulation of Microglial Cell Fcγ Receptor Expression Following Viral Brain Infection
Source: Sci Rep. 2017 Feb 6;7:41889. doi: 10.1038/srep41889 (PMC5292951; doi:10.1038/srep41889)
Supplement: Supplementary Figures and Table [file srep41889-s1.pdf]

Modulation of Microglial Cell Fcγ Receptor Expression Following Viral Brain Infection

Priyanka Chauhan, Shuxian Hu, Wen S. Sheng, Sujata Prasad, James R. Lokensgard*

Affiliation (for all authors):
Neurovirology Laboratory, Department of Medicine, University of Minnesota Medical School, Minneapolis, Minnesota (USA)

***Corresponding Author**
James R. Lokensgard
Neurovirology Laboratory, Department of Medicine, 3-107 MRF, 689 23rd Ave. S.E., Minneapolis, Minnesota (USA) 55455-1507
612-626-9914
E-mail: loken006@umn.edu

Supplementary Information

Supplementary figure 1: mRNA expression of pro-inflammatory and anti-inflammatory markers in brains of uninfected and MCMV-infected mice. RNA from uninfected and infected brain tissues of C57BL/6 mice was extracted using TRIzol reagent at 0, 5, 30, and 60 dpi and cDNA was synthesized from 1 µg of total RNA. Fold change in mRNA expression of (A) IFNγ, (B) MHC-II, (C) IL-4 and (D) TGFβ relative to uninfected control (d 0) was quantified using the $2^{-\Delta\Delta Ct}$ method and normalized to the housekeeping gene HPRT. Data shown are pooled values of two experiments using 4 mice/experiment. Data were analyzed using one-way analysis of variance (ANOVA) with Tukey's multiple comparison Test (***, $p < 0.001$; *, $p<0.05$).

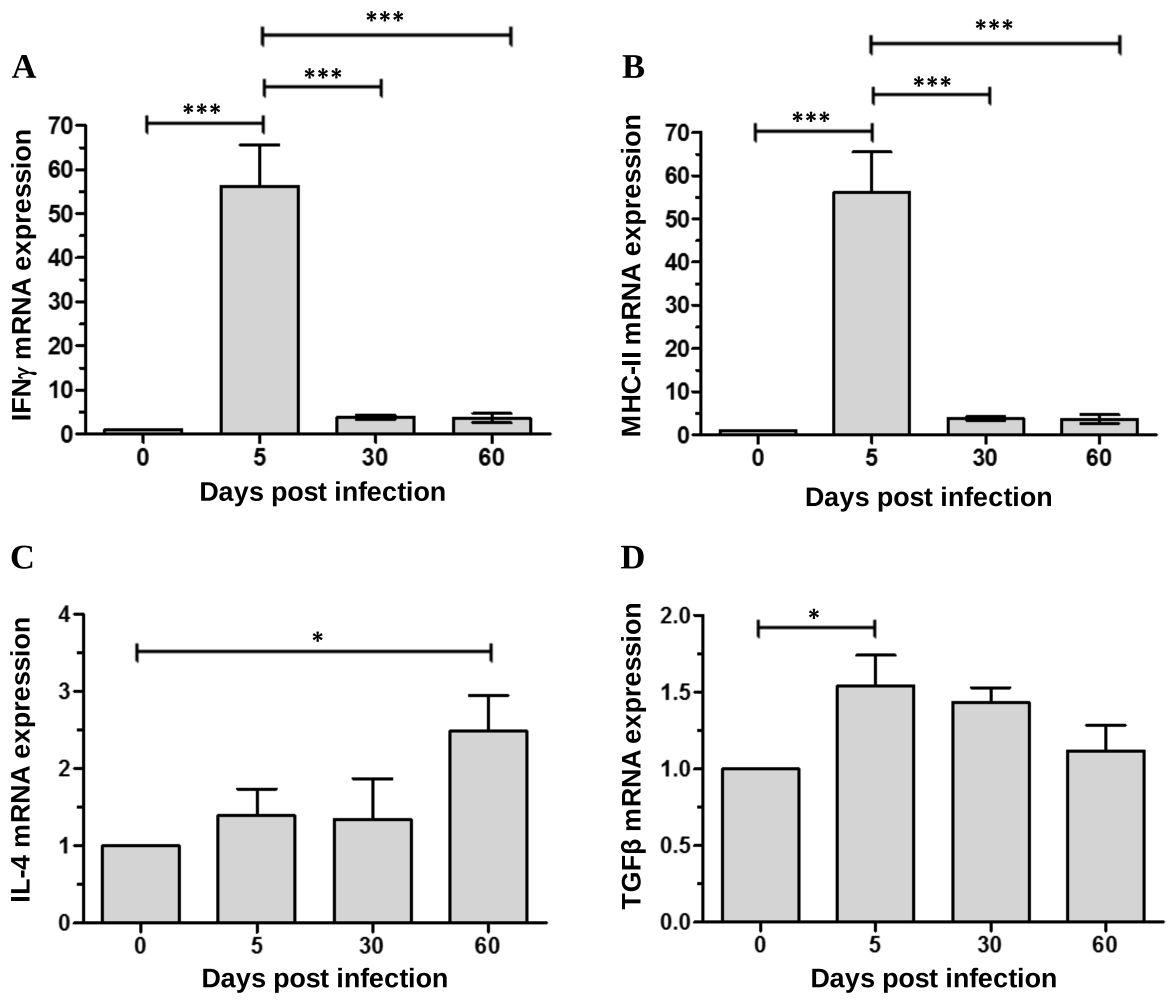

Supplementary figure 2: *in vitro* microglial cell polarization following IFNγ and IL-4 treatment. Primary murine microglial cell cultures were either unstimulated (C) or stimulated with IL-4 or IFNγ for 6 h and 24 h. cDNA synthesized from 1 μg RNA was amplified and data are expressed as fold change in mRNA expression of the indicated M1/M2 markers (A) iNOS; (B) TNF-α; (C) CD86; (D) Arginase-1; (E) E-Cadherin and (F) CD206; relative to unstimulated control (quantified using the $2^{-\Delta\Delta Ct}$ method and normalized to the housekeeping gene HPRT). Data shown are representative of two independent experiments.

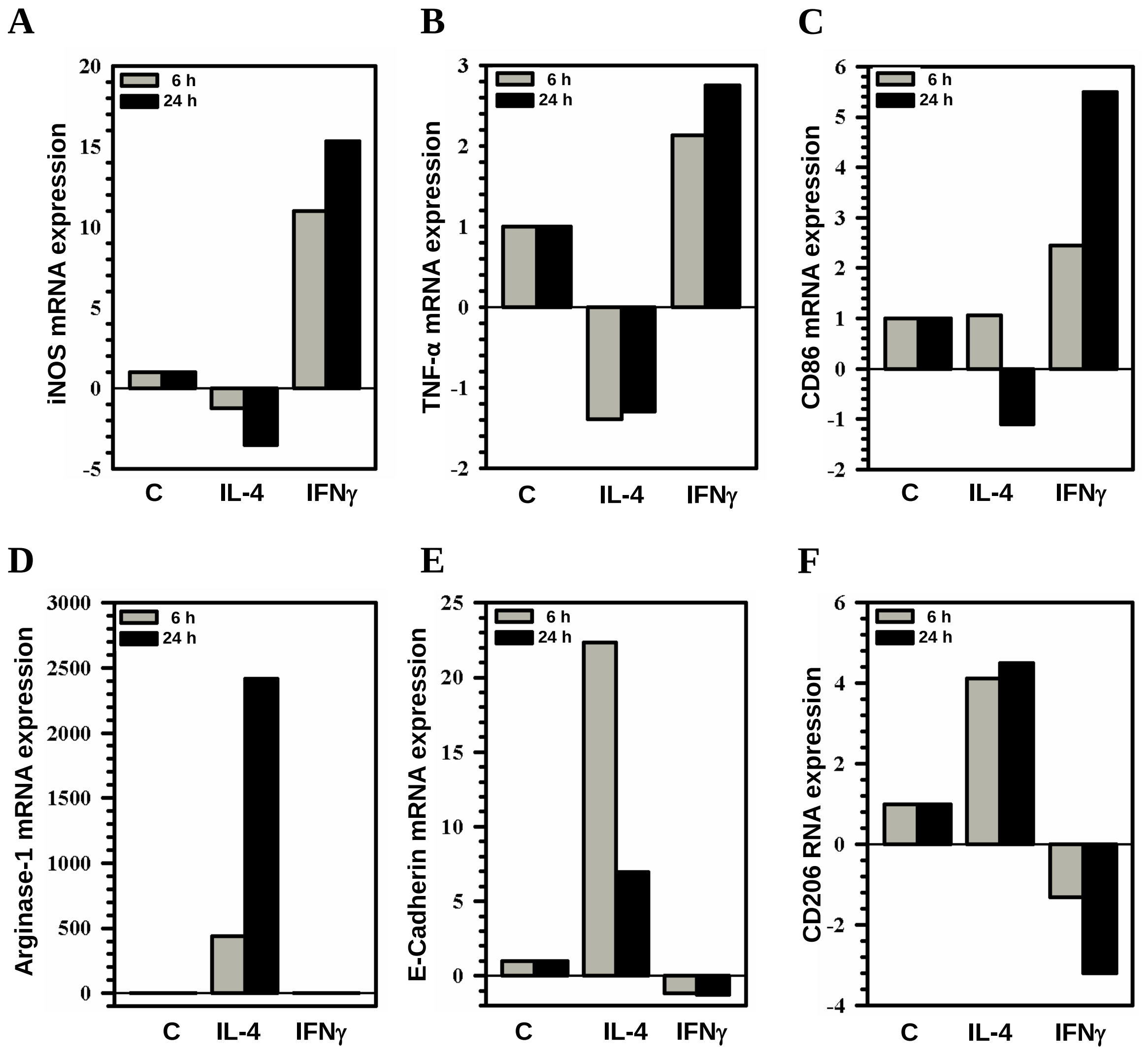

Supplementary figure 3: mRNA expression of viral IE-1 and E-1 in brains of MCMV-infected mice. RNA from uninfected and infected brain tissues of C57BL/6 (WT), Fcer1g KO, and FcgR2b KO mice was extracted using TRIzol reagent at 5 dpi and cDNA was synthesized from 1 µg of total RNA. Fold change in mRNA expression of IE-1 and E-1 relative to uninfected control (UI) was quantified using the $2^{-\Delta\Delta Ct}$ method and normalized to the housekeeping gene HPRT. Data shown are pooled values of two experiments using 4 mice/experiment. The data was analyzed using one way analysis of variance (ANOVA) with Tukey's multiple comparison Test (differences between groups were non-significant, $p > 0.05$).

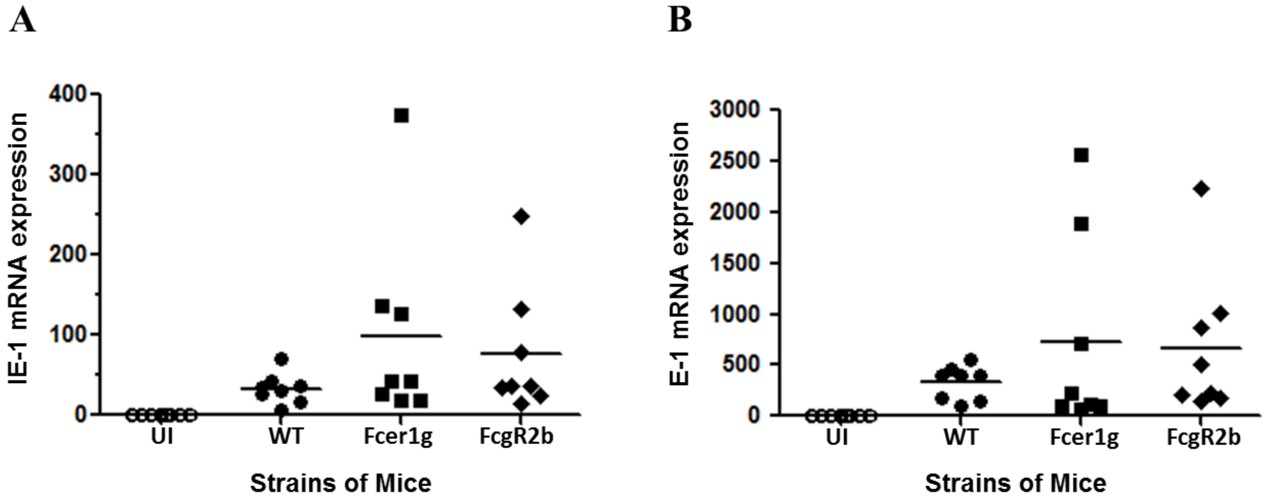

Supplementary Table 1. List of mice strains, virus, cell culture and primers used in this study.

Virus/ Experimental animals Cell line/Primers	Description	Reference
**Viral strain**		
RM461	A recombinant MCMV expressing *E. coli* β-galactosidase under the control of the human ie1/ie2 promoter/enhancer (Stoddart *et al*, 1994).	kindly provided by Edward S. Mocarski
**Experimental animals**		
C57BL/6	C57BL/6NTac	Taconic Biosciences
Fcer1g-Model 583	B6.129P2-*Fcer1g*^{tm1Rav}N12; mice deficient in the γ chain subunit.	Taconic Biosciences
FcgR2b-Model 580	B6.129S4-Fcgr2btm1TtkN12; mice deficient in FcγRIIB protein.	Taconic Biosciences
**Cell culture**		
Primary murine microglial cell culture	Microglial cells cultured from murine cerebral cortical cells from 1-day-old C57BL/6 mice	Current study
**Primers**		
F-FcγRI	*Gcggaaagagaagatgctggattc*	Current study
R-FcγRI	*cttctctctctgcagcctgtgtat*	Current study
F-FcγRIIB	*gaaaccatcacgctaaggtgcc*	Current study
R-FcγRIIB	*tggtgcagtgtccttcctagac*	Current study
F-FcγRIII	*ggtaccacactgctttctccct*	Current study
R-FcγRIII	*acttcctccagtaatccctcgg*	Current study
F-FcγRIV	*ccaccgtggcatcaaatcacat*	Current study
R-FcγRIV	*gtcctgaggttccttgctccat*	Current study
F-iNOS	tggccaccttgttcagctacg	Current study
R-iNOS	gccaaggccaaacacagcata	Current study
F-TNF-α	ctgtgaagggaatgggtgtt	Current study
R-TNF-α	ggtcactgtcccagcatctt	Current study
F-CD86	gcatatgaccgttgtgtgtgttct	Current study
R-CD86	ctctctgtcagcgttactatcccg	Current study
F-Arginase-1	gaacacggcagtggctttaac	Current study
R-Arginase-1	tgcttagctctgtctgctttgc	Current study
F-E-Cadherin	aaccagaacaaagaccaggtgacc	Current study
R-E-Cadherin	cgtagaaacagtaggagcagcagg	Current study
F-CD206	tcctgattgtgataggtgccgg	Current study
R-CD206	ctcattctgctcgatgttgccc	Current study
F-IFNγ	gcgtcattgaatcacacctg	Current study
R-IFNγ	gacctgtgggttgttgacct	Current study
F-MHC-II	gacgctcaacttgtcccaaaac	Current study
R-MHC-II	gcagccgtgaacttgttgaac	Current study
F- IL-4	cccagctagttgtcatcctgct	Current study
R- IL-4	gttcttcgttgctgtgaggacg	Current study
F-TGFβ	gcaccatccatgacatgaaccg	Current study
R-TGFβ	aaagtcaatgtacagctgccgc	Current study
